# Beta Palmitate Improves Bone Length and Quality during Catch-Up Growth in Young Rats

**DOI:** 10.3390/nu9070764

**Published:** 2017-07-18

**Authors:** Meytal Bar-Maisels, Yankel Gabet, Raanan Shamir, Sahar Hiram-Bab, Metsada Pasmanik-Chor, Moshe Phillip, Fabiana Bar-Yoseph, Galia Gat-Yablonski

**Affiliations:** 1 The Jesse Z and Sara Lea Shafer Institute for Endocrinology and Diabetes, National Center for Childhood Diabetes, Schneider Children’s Medical Center of Israel, Petach Tikva 4920235, Israel; meytalba@clalit.org.il (M.B.-M.); mosheph@post.tau.ac.il (M.P.); 2 Sackler Faculty of Medicine, Tel Aviv University, Tel Aviv 6997801, Israel; raanan@shamirmd.com; 3 Department of Anatomy and Anthropology, Sackler Faculty of Medicine, Tel Aviv University, Tel Aviv 6997801, Israel; yankel@tauex.tau.ac.il (Y.G.); saharurit@gmail.com (S.H.-B.); 4 Institute for Gastroenterology, Nutrition and Liver Diseases, Schneider Children’s Medical Center of Israel, Petach Tikva 4920235, Israel; 5 The Molecular Endocrinology Laboratory, Felsenstein Medical Research Center, Petach Tikva 4920235, Israel; 6 Bioinformatics Unit, Faculty of Life Sciences, Tel Aviv University, Tel Aviv 6997801, Israel; metsada@post.tau.ac.il; 7 Enzymotec Ltd., Sagi 2000 Industrial Park, Migdal HaEmeq 2310001, Israel; fabiana@enzymotec.com

**Keywords:** catch-up growth, μCT, biomechanics, beta palmitate, linear growth, growth plate

## Abstract

Palmitic acid (PA) is the most abundant saturated fatty acid in human milk, where it is heavily concentrated in the *sn*-*2*-position (termed beta palmitate, BPA) and as such is conserved in all women, regardless of their diet or ethnicity, indicating its physiological and metabolic importance. We hypothesized that BPA improves the efficiency of nutrition-induced catch up growth as compared to *sn*-*1,3* PA, which is present in vegetable oil. Pre-pubertal male rats were subjected to a 17 days food restriction followed by re-feeding for nine days with *1,3* PA or BPA-containing diets. We measured bone length, epiphyseal growth plate height (EGP, histology), bone quality (micro-CT and 3-point bending assay), and gene expression (Affymetrix). The BPA-containing diet improved most growth parameters: humeri length and EGP height were greater in the BPA-fed animals. Further analysis of the EGP revealed that the hypertrophic zone was significantly higher in the BPA group. In addition, Affymetrix analysis revealed that the diet affected the expression of several genes in the liver and EGP. Despite the very subtle difference between the diets and the short re-feeding period, we found a small but significant improvement in most growth parameters in the BPA-fed rats. This pre-clinical study may have important implications, especially for children with growth disorders and children with special nutritional needs.

## 1. Introduction

Human milk is considered the gold standard in infant feeding [1]. While the benefits of milk and its products are generally well-established in infants, a growing number of studies focused on the positive effects of dairy products and milk proteins on linear growth in children. In recent years researchers focus on the question of what specifically makes milk so effective (see review [2]). In addition to the immunomodulatory components (nucleotides, prebiotics, oligosaccharides, and probiotics) and other essential components with metabolic and other physiological functions (polyunsaturated fatty acids, carnitine, choline, taurine, minerals, and vitamins), the fatty components of human milk and its substitutes are drawing increasing interest, also reflecting their importance later in life [3].

Fat in human breast milk provides newborns with about 50% of the energy required for development and growth. About 98% of lipids in human milk consist of triglycerides; namely, mixtures of three fatty acids bonded to the *sn-1*, *sn-2*, or *sn-3* positions of the glycerol backbone. Palmitic acid (PA, C16:0) is the major saturated fatty acid in human milk, accounting for 17% to 25% of the total fatty acids, and is heavily concentrated at the *sn-2*-position (70–75%) [4]. Oleic acid (C18:1*n*-9), the major unsaturated fatty acid, is mostly esterified at the *sn-1* and *sn-3* positions. Unlike the other fatty acids in human milk, the *sn-2* position of palmitic acid (beta palmitate, BPA) is conserved in all women, regardless of their diet or ethnic background, which indicates important physiological and metabolic implications [5].

Milk fat is digested by bile-salt-stimulated lipase and by pancreatic lipase in the intestine. This process releases free fatty acids and 2-monoacylglycerol, which subsequently form micelles with biliary acids and are quickly absorbed [6]. Several studies have shown that PA is best absorbed in the form of BPA and is conserved as such through digestion, absorption, and chylomicron triacylglycerol synthesis [7,8,9]. When PA is esterified at the *sn-1* and *sn-3* positions, its digestion leads to the production of free PA, which tends to create complexes with dietary minerals such as calcium to form fatty acid soaps [10]. As a result, both calcium and fatty acids are lost in the stool. According to clinical studies in preterm and term infants as well as preclinical animal models, enriching infant formula with BPA results in increased fat [11] and calcium absorption [12,13], reduced calcium soap formation, and stool hardness [14,15,16], probably leading to less crying [17,18], improved bone quality [14,19], and an improved gut microbiota profile [20,21]. As a growing body of evidence has suggested the positive effects of dairy products and milk on linear growth in children and because BPA concentration is conserved in human milk, we hypothesized that BPA directly affects the growth plate and thus plays a role in linear growth.

Skeletal linear growth is driven by chondrocytes of the cartilaginous growth center in long bones, termed the epiphyseal growth plate (EGP). It is controlled by complex interactions among hormones, local growth factors, and components of the extracellular matrix (ECM). The process begins with the proliferation of resting early chondrocytes located at the most epiphyseal end of the EGP, followed by their extensive proliferation and alignment in columns parallel to the long axis of the bones. Thereafter, the cells enlarge, become hypertrophic chondrocytes with high secretory activity, and finally undergo programmed cell death or trans-differentiation to osteoblasts [22,23] with calcification of the ECM, leading to the replacement of the cartilage scaffold with bone tissue.

In children, periods of growth attenuation are usually followed by compensatory catch-up (CU) growth, defined as ‘height velocity above the normal statistical limits for age and/or maturity during a defined period of time, following a transient period of growth inhibition’ [24] that returns them to their original, genetically determined, growth trajectory. However, in many cases, depending on the child’s age, chronic illnesses, the extent of the growth deficit, and other unknown factors, CU growth is insufficient and a permanent growth deficit remains. One means of achieving more efficient CU growth may be dietary supplements. Our previous study showed nutritional intervention to be feasible, effective, and safe for promoting physical growth in short and lean pre-pubertal children [25,26].

Although the association of nutrition with linear growth is well known, the exact elements of nutrition that are necessary for growth have not been fully elucidated [27,28]. Linear growth is an excellent example of the fact that the utilization of food is not solely dependent on calories or the protein/fat ratio but also on the complex organization and configuration of the individual proteins or fatty acids. We recently reported on the effect of the different milk proteins on CU growth [29], and in the present study we investigated if BPA can affect linear growth during CU growth. The study was conducted in an animal model established in our laboratory [30], in which pre-pubertal male rats were subjected to food restriction followed by re-feeding in order to establish reversible growth attenuation and CU growth.

## 2. Materials and Methods

### 2.1. Animals and Feeding Regimens

Experiments were performed on pre-pubertal male Sprague Dawley rats weighing 50 g on average (purchased from Envigo Laboratories Ltd., Jerusalem, Israel). All animals were maintained under the same experimental conditions; mean ambient temperature 22 ± 1 °C, mean relative humidity 50 ± 2%, 12 h light/dark cycle from 6 a.m. to 6 p.m., with free access to tap water. The animals were housed individually to allow the monitoring of food intake. Before the start of the experiments, animals were given three days to become accustomed to the solitary conditions, and the experiment began at the age of 23 days. All experiments were conducted at the animal care facility of the Felsenstein Medical Research Center in accordance and with the approval of the Institutional Animal Care and Use Committee of Tel Aviv University.

The diets used in this study were provided by Teklad (South Easton, MA, USA). One diet contained PA mostly in the *sn-1*, *sn-3* configuration (Control diet-CD, TD140107), while the other, which we called the Infat Oil (IO) diet to match the commercial name of Enzymotec, Ltd. (Enzymotec, Ltd., Migdal HaEmeq, Israel) contained PA mostly in the *sn-2* position (TD140108). All other ingredients (corn starch, sucrose, cellulose, oil, and vitamins and minerals) were identical in both diets. The contents were confirmed and approved by Enzymotec Ltd. (Migdal HaEmeq, Israel) upon arrival (Table S1).

### 2.2. Procedure

In a preliminary experiment, the tolerability of the diets was tested in two groups of individually housed Sprague Dawley rats (*n* = 10 each) fed either the CD or IO diet *ad libitum* for 30 days. The animals were housed in separate cages and allowed unrestricted feeding with either the CD or IO diets for 30 days; the animals were closely monitored, and none showed any sign of disease.

Next, to test whether the study diets have differential growth-stimulation effects during CU growth, all rats were initially fed normal rat chow (2018Sc, 3.2 kcal/h; see Table S1) on a restricted protocol (60% of the normal daily intake) for 17 days. The 40% restriction was calculated based on previous studies in which animals were housed individually, and the amount of food consumed each day was measured together with the animal’s weight and weight gain [30]. Body weight was measured two to three times weekly. On day 17, the rats were randomly allocated to receive the CD or IO diet (*n* = 8 each) for unrestricted re-feeding for 9 days. On day 26 of the experiment, the rats were killed by CO_2_ inhalation. The animals were observed daily throughout the study, and all remained healthy with no evidence of gastrointestinal disorder. The experiment was terminated when the rats were 49 days old in order to avoid the confounding effects of sex hormones [31], as the surge in sex hormones, particularly estrogen, causes shrinkage of the EGP and growth cessation (in humans) or attenuation (in rats) [32,33].

At sacrifice, blood was collected by cardiac puncture, and the serum was separated and kept at −20 °C until analyzed. The internal organs were removed, weighed, and stored at −70 °C. Tibiae and humeri were carefully cleaned of soft tissue and humeri were measured with a digital caliper, and humeri were prepared for further analysis. The EGPs were collected from the tibias, snap-frozen in liquid nitrogen, and kept at −70 °C.

### 2.3. Serum Analysis

Serum was separated by centrifugation at 450 g for 10 min at 4 °C and stored at −70 °C. The chemical analysis of the serum was performed by American Medical Laboratories (AML) Ltd. (Herzlia, Israel). Serum levels of insulin-like growth factor-1 (IGF-1) and leptin were measured using commercial kits according to the manufacturer’s recommendations: Quantikine Mouse/Rat IGF-1 assay kit, detection limit 8.4 pg/mL (Cat. No. MG100, R & D Systems, Minneapolis, MN, USA); Rat Leptin ELISA kit, detection limit 22 pg/mL (Millipore, Billerica, MA, USA).

### 2.4. Measurement of Crude Liver Lipid Content (Folch Method)

Chloroform/methanol at a relative volume of 2:1 was added to 100 mg of homogenized liver tissue. The liquid phase was washed with water and centrifuged, and the lower phase was air-dried, weighed, and calculated per 100 mg of tissue [34].

### 2.5. Histological Staining and Measurement of EGP Height

The cleaned humeri (one per rat) were fixed in 4% neutral buffered formalin (NBF) for 48 h at room temperature, decalcified with Ethylenediaminetetraacetic acid (EDTA) and hydrogen chloride (Calci-Clear Rapid, Cat. No. HS-105, National Diagnostics, Atlanta, Georgia) for 7 h, dehydrated through graded ethanol series (70%, 95%, 100%), and stabilized by two sequential changes of chloroform for paraffin embedding. A general histomorphological evaluation was performed on deparaffinized sections stained with hematoxylin-eosin and Alcian blue. EGP height was measured from the reserve zone to the ossification front of the metaphyseal bone in stained paraffin sections of 5 μm thickness. The slides were photographed under an Olympus BX40 microscope equipped with an Olympus DP71 camera (Olympus Optical Co. GmbH, Hamburg, Germany) and analyzed using Image-Pro software (version 4.5.1.22, Media Cybernetics, Inc., Rockville, MD, USA).

### 2.6. RNA Extraction and Affymetrix Analysis

Total RNA was extracted from the liver and EGP tissues using the miReasy Mini Kit (Cat. No./ID: 217004, Qiagen, Valencia, CA, USA) according to the manufacturer’s protocol (*n* = 4 in each group). The quantity and quality of the RNA were evaluated using a Nanodrop spectrophotometer (Thermo Scientific Corp., Wilmington, DE, USA) and an Agilent 2100 Bioanalyzer (Agilent Technologies, Santa Clara, CA, USA) with values of A260/A280 >2.0 and A260/A230 >1.7 and RNA integrity number (RIN) >6.1. Equal amounts of RNA were used for the analysis by the Rat Affymetrix Gene Chip expression array (Rat Gene 2.X ST, Affymetrix, Thermo Scientific). The whole procedure was performed at the Functional Genomics Unit of Tel Aviv University. Quality control approved all samples. For internal quality control, we confirmed the differential expression of a panel of genes known to be specific for each of the tissues (e.g., CPS-1 for the liver [35] and collagen types II and X for the EGP). Clustering and analysis of variance (ANOVA) calculations were done using the Partek Genomics Suite, v 6.6 (Partek Inc., St. Louis, MO, USA) 

### 2.7. Reverse Transcription and Real Time PCR for GDF-5

To measure the expression of growth and differentiation factor 5 (GDF-5) in EGP, first-strand cDNA synthesis was performed with the PrimeScript First-Strand cDNA Synthesis Kit (Takara Bio, Mountainview, CA, USA) using 1 μg of the total RNA as a template, according to the manufacturer’s instructions. A real-time quantitative polymerase chain reaction (qPCR) was performed with the ABI Prism 7000 Sequence Detection System (Applied Biosystems Inc., Foster City, CA, USA), according to the manufacturer’s instructions and using specific FAM- labeled probes (TaqMan^®^ assay on demand Rn0043356-m1 for GDF5); Rn00667086 for Aco2 served as the internal control [30]). The following thermal cycling conditions were used; one cycle at 50 °C for 2 min and at 95 °C for 10 min, followed by 45 cycles of 15 s at 95 °C and 1 min at 60 °C. The probes, reaction mixture, and 7000 Sequence Detection System were all obtained from Applied Biosystems. Relative expression was determined using the 2^−ΔΔ*C*t^ method. Each sample was examined in triplicate.

### 2.8. μCT Analysis

Humeri were maintained in 4% NBF for 48 h at room temperature and then stored in 70% ethanol. Whole humeri (one per rat) were scanned using a micro-computed tomography (μCT) system (μCT50, Scanco Medical AG, Brüttisellen, Switzerland). Scans were acquired at 90 kVp, 200 μA, and 1000 ms for energy, intensity, and integration time, respectively, generating images with an isotropic nominal resolution of 17.2 μm. Two-dimensional CT images were reconstructed in 2048 × 2048 pixel matrices using a standard convolution-back projection procedure (Scanco μct_reconstruction version 6.1; Scanco Medical AG, Brüttisellen, Switzerland). A three-dimensional Gaussian filter was used to attenuate the background noise (σ = 0.8; support = 1). The scans were segmented using a global thresholding procedure (trabecular attenuation = 130; cortical attenuation = 200; in permille of the total gray value range). The morphometric parameters were determined by a direct three-dimensional approach in three different pre-selected regions using customized software developed on the proprietary Image Processing Language version v5.15 (Scanco). In the whole bone, we measured length and full volumetric bone mineral density (vBMD). In the cortical bone, we used a 1-mm-height diaphyseal segment starting at the 6th tenth of the total length (slightly distal to the midshaft) distal to the deltoid tuberosity. The cortical measurements included total area (Tt.Ar, mm^2^), cortical area (Ct.Ar, mm^2^), cortical area fraction (Ct.Ar/Tt.Ar, %), and cortical thickness (Ct.Th, mm). To analyze the trabecular bone, we used the secondary spongiosa of the proximal metaphysis of the humerus, separated manually from the cortical bone. The trabecular bone region of interest (tROI) started at the distal tip of the primary spongiosa and extended 3.44 mm distally. This ROI was further divided into two 1.72 mm proximal (pROI) and distal (dROI) halves to depict region-specific changes. The measurements included the bone volume fraction (BV/TV), trabecular number (Tb.N, mm^−1^), trabecular thickness (Tb.Th, mm), and trabecular separation (Tb.Sp, mm). All parameters were analyzed according to standardized guidelines and nomenclature [36].

### 2.9. Biomechanical Analysis

Twenty-four hours before biomechanical testing, humeri were rehydrated in phosphate-buffered saline to restore the mechanical properties of the tissue [29,37,38,39]. The three-point bending test was performed using a loading machine (Bose ElectroForce 5500, TA Instruments, New Castle, DE, USA) equipped with a 200 N load cell. The bone specimens were placed in a custom-made device with a span length of 20 mm, with the middle probe located at the same diaphyseal segment analyzed by μCT. Specimens were loaded until failure at a cross-head speed of 1 mm/min, and force versus displacement data were acquired automatically. The load-displacement curve yielded three parameters for evaluation: bending stiffness (N/mm), calculated as the slope of the load-deflection curve at its linear portion; ultimate/maximal load (N); and energy to maximal load (N*mm), calculated as the area under the curve (AUC).

### 2.10. Statistical Analysis

Data are presented as mean ± standard deviation (SD). The significance of differences between experimental groups was determined with Student’s *t*-test. Differences were considered statistically significant at *p* < 0.05. SPSS v23 software (IBM, Armonk, NY, USA) was used for statistical analysis of the data.

## 3. Results

### 3.1. Effect of the CD and IO Diets on Weight Gain and Serum Values in the Preliminary Experiment

The preliminary experiment in which animals were fed ad libitum showed that both the Control Diet (CD) and the Infat Oil diet (IO) were very well tolerated by the rats, with no adverse effects. Weight and weight gain at the termination of this first experiment were similar in the two groups (and similar to those of rats fed the normal chow diet [29]), and there were no significant between-group differences in the weight of the internal organs (liver, heart, lungs, and kidneys), humerus length, or humeri EGP height (Table S2). Chemical analysis of the serum showed that all values, including IGF-1 and leptin, were within normal range for Sprague-Dawley rats, with no significant differences between the groups (normal range provided by AML Ltd.; leptin and IGF-I levels were similar to those of previous experiments [29]). Calcium and triglyceride levels were above the normal range, but they did not differ between the two groups (Table S3).

### 3.2. Effect of the Study Diets on Weight Gain and Serum Values during CU Growth

On the refeeding experiment, after dietary restriction, body weight and weight gain were similar in the two groups, as were liver weight and total fat content (Table 1). Chemical analysis of the serum showed that all values were within the normal range, including IGF-1 and leptin (Table 1), with no significant differences among the groups (Table S4).

### 3.3. Effect of the Study Diets on Linear Growth during CU Growth

**Figure 1:**
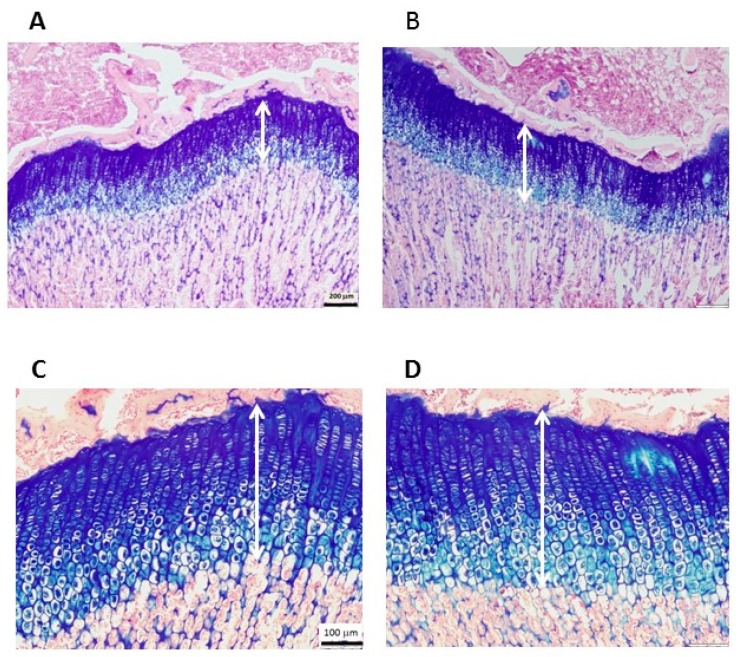
Representative stained sections of the epiphyseal growth plate (EGP) of Sprague Dawley rats, 49 days old. Hematoxylin and eosin and Alcian blue staining shows the margins of the cartilaginous EGP. (**A**,**C**) Control Diet; (**B**,**D**) Infat Oil diet. (**A**,**B**) show a magnification X4, scale bar = 200 μm; (**C**,**D**) show a magnification X10, scale bar = 100 μm; six sections were measured in each group.

Despite their similar body weight after the refeeding period, rats given the IO diet showed improved growth parameters. Humerus length was higher in IO group relative to the CD group (Table 1; *p* = 0.042). Animals fed the IO diet showed a trend toward a 5% higher EGP than the CD-fed rats (*p* = 0.06, Figure 1). Further analysis of the EGP revealed that the greater height was due to an increase in both the proliferative and hypertrophic zones, with the hypertrophic zone being significantly higher in the IO than the CD group (*p* = 0.02). The EGP morphology remained intact (Figure 1), indicating that there were no adverse effects from any of the diets.

### 3.4. Effect of the Study Diets on Bone Quality during CU Growth

**Figure 2:**
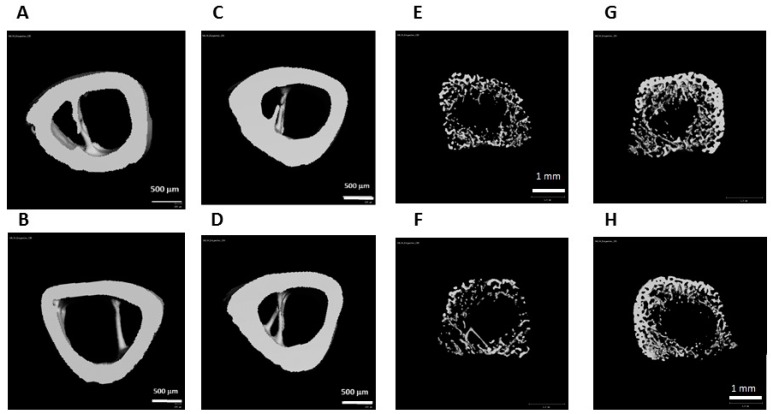
Three-dimensional cortical (left) and trabecular (right) bone images obtained by µCT. (**A**,**B**,**E**,**F**) Control Diet; (**C**,**D**,**G**,**H**) Infat Oil diet (Two representative figures are presented per each group). The calculations were done on the cortical ring only, without the trabeculae.

Bone microstructure and quality were evaluated by μCT and biomechanical testing. Cortical thickness (Ct.Th) was significantly higher in the IO group (Table 2; Figure 2; *p* = 0.026), and the ratio of cortical area (Ct.Ar) to total area (Tt.Ar) tended to be higher in the IO group (*p* = 0.058).

In the trabecular bone compartment, all values (BV/TV, Tb.Th, and Tb.N) seemed to be higher in the IO group, but the difference did not reach statistical significance. Only when the distal half of the proximal metaphysis (dROI) was analyzed separately, were the difference in the BV/TV values statistically significant.

Interestingly, although the results were not statistically significant, on three-point bending analysis, the IO group showed a lesser bone stiffness and higher energy to maximal force.

### 3.5. Effect of the Different Diets on Gene Expression during CU Growth

To study the molecular mechanism underlying the differences in linear growth as well as bone microstructure and quality among the groups, we performed gene expression analysis on liver and EGP samples. Only a small number of genes were found to be differentially expressed among the groups.

In the liver, changes were found mainly in metabolic genes (annotation ‘metabolism’ according to the David website (https://david.ncifcrf.gov/). Of the 112 metabolic genes that were significantly different between the two diet groups, none has been previously associated with PA metabolism. The 20 genes that were most affected are presented in Table S5. Three genes, *Nampt*, *Alas1*, and *Mllt3*, were significantly higher (by >1.5 fold) in the IO than the CD group; gene expression levels for *Egr1*, *Rnf125*, and *Lox* were significantly lower in the OI group.

**Figure 3:**
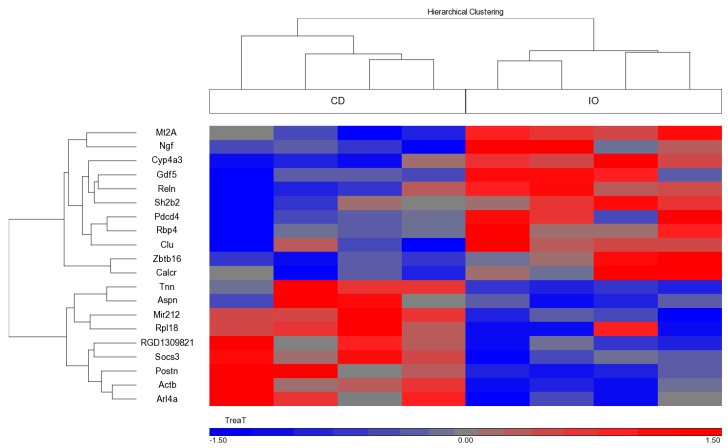
Differential gene expression in the EGP according to Rat Affymetrix Gene Chip expression array (Rat Gene 2.X ST, Affymetrix, Thermo Scientific) (*n* = 4 per each group). Heatmap of the genes that were differentially expressed in EGP in the Infat Oil (IO) versus the Control Diet (CD) fed animals (*p* < 0.05 and FC = 1.5). The heatmap was created using Partek GS.

A similar analysis performed on RNA extracted from the EGP showed that most of the known cartilage-specific genes were not significantly affected, although some tended to be more highly expressed in samples from the IO than the CD group. Of the genes with a significant differential expression, the 20 that were most affected are shown in Table 3 (and Figure 3). Of those known to be associated with growth, four were upregulated in the IO group, namely, *Mt2a*, *Rbp4*, *Ngf*, and *Gdf-5*, and four were downregulated, namely, *Aspn*, *Tnn*, *Postn*, and *Soc-3*.

Since we previously showed that growth differentiation factor-5 (Gdf-5), was associated with both fat and growth regulation [40], we evaluated the RNA transcript levels of this gene by qPCR. A similar tendency for a higher Gdf-5 expression in the IO group was noted, but the results were not statistically significant (data not shown).

## 4. Discussion

Observational studies conducted mainly in deprived geographic regions have highlighted several nutritional components that are required for proper growth, including macronutrients like proteins, lipids, and carbohydrates and micronutrients like minerals and vitamins. However, can we also affect the efficiency of the growth process in the presence of sufficient basic nutrition? This study and our previous one [29] suggest that we can. In the current study, we show that a subtle change, the esterification position of fatty acid, may also affect bone elongation and quality.

Our data show that a modification as seemingly minor as a shift in the ratio of BPA to *sn-1,3* PA, significantly affected EGP height, bone thickness, strength, and even gene expression in the liver and EGP. The differences in most of the parameters were small, as expected by the very subtle change we imposed on the system, but all supported the advantage of BPA over *sn-1,3* PA. Thus, different diets may affect the microstructure and quality of the growing skeleton even when they are matched in composition for calories as well as macro- and micro-nutrients, at least in the short term.

In our previous studies, 40% food restriction in young, weaned rats led to a significant reduction in total body weight, bone, and EGP lengths, as well as bone quality [29,41]. Subsequent nutrition-induced CU growth was associated with a rapid increase in weight, EGP height, bone length, and bone quality. The degree of improvement was time-dependent [29]. In the present study, although we did not find differential effects on bone length and EGP height under baseline conditions (as in the preliminary experiment), there were significant differences when the diets were given after a period of food restriction. The best explanation for this discrepancy may be that as *ad libitum* feeding is already above the normal food requirements [35], a loss of fat and calcium will have no effect as they are already in excess. However, after an insult of food restriction, the body requires all the calcium and fat that are available, and even marginally sub-optimal nutrients can affect the efficiency of bone growth.

The increase in bone length and EGP height in the IO diet group point to the beneficial effect of dietary BPA on linear growth, as a higher yet organized EGP may suggest better growth potential. Despite the greater bone length, bone quality was not hampered, indicating that enough calcium was obtained from the diet for proper mineralization of the nascent bone, and resistance to fracture was enhanced. These observations are supported by previous data on the effect of BPA on calcium absorption [11,13], but they may also suggest a better endochondral ossification process (in the metaphysis) and bone apposition (in the mid-diaphysis), leading to higher EGP and stronger bones, respectively. The tendency to lesser bone stiffness in the IO groups relative to the CD group suggests that the bone midshaft was slightly less brittle. Since the maximal force was similar, the resulting displacement to failure was extended, indicating a tendency for the bones of the IO-fed rats to be more resistant by being more flexible (or pliable) and therefore absorb more energy before failure.

In the serum analysis, we measured two systemic factors, leptin and IGF-1, previously shown to be most affected by diet. IGF-1 concentrations are responsive to changing nutritional status and the intake of amino acids and free fatty acids [42,43,44]. Leptin is associated with obesity and is used as a surrogate marker of energy level. We found that re-feeding increased the levels of IGF-1 and leptin to a similar extent in both groups, and the values were within the normal range. IGF-1 circulates in the plasma tightly bound to specific binding proteins (IGF-BP) and to the acid labile subunit (IGF-ALS), which stabilizes IGF-I and extends the serum half-life of IGF-I [35]. No differences were observed in the expression levels of IGF-1, IGF-BPs, or IGF-ALS in the liver, and, as the liver is the major organ synthesizing and secreting these factors, it is reasonable to assume (although it was not tested) that the level in the blood was also unaffected.

An analysis of gene expression in the liver and EGP yielded, as expected, a very low number of affected genes. In the liver, most of those were genes controlling metabolic enzymes, in line with the metabolic function of the liver. Only three genes, *Nampt*, *Alas1*, and *Mllt3*, showed a significantly greater increase (by more than 1.5-fold) in the IO group than the CD group. Although a literature search to determine the function of these genes failed to identify any known association with linear growth, these genes may still be associated with growth. *Nampt* (nicotinamide phosphoribosyl transferase) encodes a protein that catalyzes the condensation of nicotinamide with 5-phosphoribosyl-1-pyrophosphate to yield nicotinamide mononucleotide, an intermediate agent in the biosynthesis of nicotinamide adenine dinucleotide (NAD) [45]. The association of this with growth may reside in the critical role apparently played by *Sirt1* and *Nampt* in regulating insulin sensitivity and secretion throughout the body [46] and the reported effect of nutrition on *Sirt1* expression [44,47]. *Alas1* (5′-aminolevulinate synthase 1) encodes the mitochondrial enzyme that catalyzes the rate-limiting step in heme biosynthesis and was reported to be involved in the regulation of lipid metabolism by peroxisome proliferator-activated receptor alpha (PPARα) [48]. *Mllt3* (myeloid/lymphoid or mixed-lineage leukemia; translocated to 3) encodes a component of the complex required to increase the catalytic rate of RNA polymerase II transcription, which we found to be significantly increased during re-feeding. However, on a proteomic analysis of the liver, the entire transcription machinery, and not just one gene, was found to be upregulated [35].

Three genes for which expression was lower in the liver samples from the IO compared to the CD group were *Egr1*, *Rnf125*, and *Lox*. *Egr1* (early growth response 1) encodes a zinc finger protein that functions as an early transcriptional regulator responsive to cues of differentiation and mitogenesis such as changes in insulin and IGF-I [49]. *Rnf125* (ring finger protein 125) encodes E3 ubiquitin ligase, which mediates the ubiquitination and subsequent proteasomal degradation of target proteins. Several mutations in *Rnfl25* were recently reported in patients with overgrowth syndrome, pointing to its importance in growth regulation [50]. *Lox* (Lysyl oxidase) encodes an extracellular amine oxidase that functions in the crosslinking of collagens and elastin. In the liver, it is mostly associated with fibrosis [51], but, in osteoblasts, the organization of the collagen fibers in the extracellular matrix is an important regulator of osteoblastogenesis [52].

In the EGP, we found a different battery of affected genes, grouped under the classification of ‘growth and development’. Several interesting genes were upregulated in the IO fed rats, including *Mt2a*, *Rbp4*, *Ngf*, and *GDF5,* and several were downregulated, including *Aspn*, *Tnn*, *Postn*, and *Soc3*. *Mt2a* (metallothionein 2A) is affiliated with the lncRNA class. *Rbp4* (retinol binding protein 4) belongs to the lipocalin family and is the specific carrier for retinol (vitamin A alcohol) from the liver stores to the peripheral tissues. The Drosophila orthologue of *Rbp4*, Neural Lazarillo (*NLaz*), was found to be a secreted protein that suppresses insulin signaling [53]. Furthermore, NLaz mutant flies were bigger in size; however the process by which it negatively regulates larval growth in normal nutritional conditions is still unresolved. *Ngf* (nerve growth factor) exerts nerve-growth-stimulating activity. The level of the Ngf protein receptor is regulated by growth hormone, suggesting a cross-talk of Ngf with the growth-regulating process [54]. A downregulated gene, *Aspn* (asporin) encodes a class I small leucine-rich proteoglycan (SLRP) that is expressed in very low levels in the EGP and was shown to be responsive to different cytokines in human articular chondrocytes and involved in osteoarthritis [55]. *Tnn* (tenascin N) encodes a protein involved in the degradation of ECM and in miRNA regulation [56]. *Postn* (Periostin) encodes a protein secreted by the ECM that functions in tissue development and regeneration by binding to integrins and heparin in the ECM to support the adhesion and migration of cells. The Postn protein enhances the incorporation of bone morphogenic protein (BMP)-1 into the fibronectin matrix of connective tissues and the subsequent proteolytic activation of Lox, and it serves both as a structural molecule of the bone matrix and a signaling molecule through integrin receptors and Wnt-beta-catenin pathways, whereby it stimulates osteoblast functions and bone formation [57]. *Socs* (suppressor of cytokine signaling)-3 encodes a member of the Signal transducer and activator of transcription (STAT)-induced STAT inhibitor (SSI) and cytokine-inducible negative regulators of cytokine signaling, including gp130, LIF, erythropoietin, insulin, IL12, G-CSF, and leptin receptors.

The increase in *Gdf-5* in the IO compared to the CD group is interesting, as a recent study by our group revealed that GDF-5 is produced and secreted by adipocytes in culture and stimulates the growth of metatarsals in vitro. Its level was also increased under conditions of nutritional CU growth in vivo [40]. The induction of GDF5 by the IO diet may explain the beneficial effect of the diet on EGP height. *GDF5* is a member of a subfamily of the highly conserved group of BMPs. It is active during mesenchymal cell condensation, initiating the first stages of chondrogenesis by promoting cell adhesion [58]. It may also increase the size of skeletal elements [58], stimulate proteoglycan production in chondrocytes [59], and serve as a positive regulator of bone healing [60,61,62]. In a large multinational genetic study, a locus near the GDF5 gene on chromosome 20 was found to be associated with final height in humans [63].

The changes in the above mentioned genes, although small, were statistically significant by Affymetrix analysis and may point to their involvement in the growth response.

The present study shows that the conformation of dietary PA has a significant effect on skeletal growth. This may be due, as previously shown, to the effect of PA on calcium absorbance (although bone marrow density and calcium serum levels were similar in the study groups) or on gut microbiota [20] or its effects on the expression of specific genes in the liver and EGP.

The main limitation of this study is our use of an animal model, which has inherent differences from a human model, including the short time period from weaning to puberty, necessitating a short time of follow up after re-feeding to avoid the involvement of sex steroids. However, animal models are of the utmost importance because they allow for relatively rapid evaluation of the efficiency of nutritional manipulation and the results may be meaningful as rats and humans are quite similar in the physiology and anatomical structures of their gastrointestinal tracts (see [64,65,66]). Another limitation is that we followed the animals for only a short period, just until puberty; further long term studies are required to identify the effect of BPA on final height and adult bone quality. In addition, the differences in gene expression were difficult to reproduce by qPCR, although a trend was noted, possibly due to the small changes.

## 5. Conclusions

We have shown in our model of nutrition induced catch up growth that despite the very subtle modification between the diets, rats fed a diet with BPA had improved EGP height and longer bones with better bone microstructure and quality. This may be due, as previously shown, to the effect of BPA on calcium absorbance or on gut microbiota or its effects on the expression of specific genes in the liver and EGP. The diet affected the expression of several genes associated with metabolism in the liver and with growth and development in the EGP. Among them the most important seem to be *Gdf-5*, known to stimulate growth, that was increased and *Socs3*, a negative regulators of cytokine signaling, including GH and leptin that was reduced. This pre-clinical study may have important implications, especially for children with growth disorders and children with special nutritional needs.

## Figures and Tables

**Table 1:** Effect of the diets on growth parameters.

Growth Parameters	Control 1,3 Diet (CD) (*n* = 8) (Mean ± SD)	Infat Oil (BPA) Diet (IO) (*n* = 8) (Mean ± SD)	*T*-Test (*p* Value)
Weight (g)	131.3 ± 7.9	135.6 ± 8.4	0.31
Weight gain (g)	83.5 ± 5.1	87.9 ± 6.4	0.17
Liver weight (g)	6.9 ± 0.3	6.8 ± 0.5	0.81
Liver fat content (mg/100 mg tissue)	4.5 ± 0.5	4.7 ± 0.7	0.64
Full humerus length (mm)	19.86 ± 0.5	20.4 ± 0.3	0.042
EGP height (mm)	0.39 ± 0.02	0.41 ± 0.02	0.06
Proliferative (mm)	0.22 ± 0.04	0.21 ± 0.03	0.44
Hypertrophic (mm)	0.18 ± 0.02	0.2 ± 0.02	0.02
Proliferative/hypertrophic	1.21 ± 0.18	1.03 ± 0.14	0.05
IGF-I (ng/mL)	868.1 ± 193.7	894.2 ± 108.1	0.78
Leptin (pg/mL)	1461 ± 196	1448 ± 434.8	0.94

**Table 2:** Effect of the diets on bone parameter values by µCT and 3-point bending assay.

Bone Parameters	Control 1,3 Diet (CD) (*n* = 8) (Mean ± SD)	Infat Oil (BPA) Diet (IO) (*n* = 8) (Mean ± SD)	*T*-Test (*p* Value)
Full volumetric bone mineral density (vBMD) (mg HA/cm^3^g)	296.7 ± 14.85	306.58 ± 15.2	0.262
(A) Cortical bone parameters			
Tt.Ar (mm^2^)	3.15 ± 0.07	3.07 ± 0.11	0.51
Ct.Ar (mm^2^)	1.75 ± 0.1	1.83 ± 0.12	0.31
Ct.Ar/Tt.Ar (%)	55 ± 0.02	59 ± 0.04	0.058
Ct.Th (mm)	0.31 ± 0.01	0.35 ± 0.03	0.026
(B) Trabecular bone parameters (tROI)			
BV/TV (%)	3.9 ± 1.31	5.26 ± 2.02	0.17
Tb.Th (mm)	0.05 ± 0.004	0.06 ± 0.01	0.11
Tb.N (mm-1)	1.06 ± 0.15	1.19 ± 0.26	0.28
Tb.Sp (mm)	0.96 ± 0.14	0.89 ± 0.15	0.41
(B1) Proximal ROI (proximal metaphysis)			
BV/TV (%)	5.7 ± 2	7.5 ± 3	0.22
Tb.Th (mm)	0.05 ± 0.004	0.06 ± 0.01	0.11
Tb.N (mm^−1^)	1.46 ^a^ ± 0.2	1.54 ± 0.29	0.55
Tb.Sp (mm)	0.71 ± 0.09	0.69 ± 0.11	0.73
(B2) Distal ROI (proximal metaphysis)			
BV/TV (%)	0.72 ± 0.15	1.25 ± 0.5	0.015
Tb.Th (mm)	0.05 ± 0.0	0.05 ± 0.01	0.98
Tb.N (mm^−1^)	0.71 ± 0.1	0.84 ± 0.21	0.154
Tb.Sp (mm)	1.42 ± 0.21	1.25 ± 0.23	0.21
(C) Bone biomechanical properties			
Stiffness (N/mm)	41.68 ± 14.4	36.37 ± 7.6	0.43
Maximal load (N)	22.8 ± 4.3	21.54 ± 3.5	0.53
Energy to maximum (N*mm)	14.02 ± 3.5	16.5 ± 5.0	0.3

Superscripts denote significant between-group differences as follows (*p* < 0.05); cortical thickness and the bone volume fraction (BV/TV) of the distal bone were significantly different. vBMD, full volumetric bone mineral density; Tt.Ar, total area; Ct.Ar, cortical area; Ct.Th, cortical thickness; BV/TV, bone volume fraction; Tb.Th, trabecular thickness; Tb.N, trabecular number, Tb.Sp, trabecular separation.

**Table 3:** Top 20 genes in the EGP significantly affected by the position of palmitic acid in the diet (all data are significant at *p* < 0.05 (ANOVA).

Gene Symbol	Gene Name	Gene ID	Fold Change (IO/CD)
Mt2A	Metallothionein 2A	NM_001137564	1.56
Rbp4	Retinol binding protein 4	NM_013162	1.5
Calcr	Calcitonin receptor	NM_001034015	1.42
Reln	Reelin	NM_080394	1.37
Clu	Clusterin	NM_053021	1.36
Sh2b2	SH2B adaptor protein 2	NM_053669	1.35
Ngf	Nerve growth factor (beta polypeptide)	NM_001277055	1.35
Pdcd4	Programmed cell death 4	NM_022265	1.34
Cyp4a3	Cytochrome P450, family 4, subfamily a, polypeptide 3	NM_175760	1.3
Gdf5	Growth differentiation factor 5	ENSRNOT00000073736	1.28
Zbtb16	Zinc finger and DTD domain containing 16	NM_001013181	1.28
Actb	Actin, beta	ENSRNOT00000034844	−1.26
Socs3	Suppressor of cytokine signaling 3	NM_053565	−1.26
Arl4a	ADP-ribosylation factor like GTPase 4A	NM_019186	−1.26
Mir212	microRNA 212	NR_031925	−1.27
RGD1309821	Similar to KIAA1161 protein	NSRNOT00000033235	−1.34
Rpl18	Ribosomal protein L18	FQ229993	−1.35
Postn	Periostin, osteoblast specific factor	NM_001108550	−1.43
Tnn	Tenascin N	NM_001107189	−1.56
Aspn	Asporin	NM_001014008	−1.72

## Supplementary Materials

The following are available online at www.mdpi.com/2072-6643/9/7/764/s1, Table S1: Macronutrient, vitamin, and mineral content of the experimental diets, Table S2: Growth parameters for the preliminary experiment, Table S3: Serum chemical analysis from the preliminary experiment, Table S4: Serum chemical analysis after re-feeding, Table S5: Top 20 metabolic genes that were affected in the liver by the position of palmitic acid in the diet. Reference [29] is cited in the supplementary materials.
