# Supplementary material for: Beta Palmitate Improves Bone Length and Quality during Catch-Up Growth in Young Rats
Source: Nutrients. 2017 Jul 18;9(7):764. doi: 10.3390/nu9070764 (PMC5537878; doi:10.3390/nu9070764)
Supplement: Supplementary file 1 [file nutrients-09-00764-s001.zip › nutrients-204807-supplementary.pdf]

Table S1. Macronutrient, vitamin, and mineral content of the experimental diets.

	2018SC (Normal Chow)	TD.140108 (Control Diet)	TD.140107 (Infat Oil Diet)
Energy (Kcal/g)	3.1	3.2	3.2
Carbohydrate (g/Kg)	442	486	486
Protein (g/Kg)	186 [a]	205.0	205
Fat (g/Kg)	60	56	56
Fiber (cellulose) (g/Kg)	147	150	150
	Minerals		
Calcium, g/kg	10	10	10
Phosphorus, g/kg	7 [b]	4	4
Sodium, g/kg	2	2	2
Potassium, g/kg	6	7	7
Chloride, g/kg	4	3.1	3.1
Magnesium, g/kg	2	1	1
Zinc, mg/kg	70	70	70
Manganese, mg/kg	100	20	20
Copper, mg/kg	15	12	12
Iodine, mg/kg	6	0.4	0.4
Iron, mg/kg	200	71	71
Selenium, mg/kg	0.23	0.29	0.29
	Vitamins		
A (IU/g)	15	15	15
B1 (thiamin), mg/kg	17	17	17
B2 (riboflavin), mg/kg	15	22	22
B3 (niacin), mg/kg	70	99	99
B6, mg/kg	18	18	18
B12, mg/kg	0.08	0.03	0.03
C [c], mg/kg	-	1	1
D3, IU/g	1.5	1.5	1.5
E, mg/kg	110	100	100
Choline, mg/kg	1200	1200	1200
Biotin, mg/kg	0.4	0.4	0.4
Folic acid, mg/kg	4	2	2
K3, mg/kg	50	50	50
Pantothenic acid mg/kg	33	60	60
	Fat		
C14	ND	0.3	0.2
C16:0	13.0	14.4	13.9
C16:0 sn-2 ratio	13.5	12.9	53.0
C16:1	ND.	0.2	0.1
C18	3.0	3.1	3.4
C18:1 n9	20.4	32.1	36.7
C18:1 n11	1.2	1.1	1.0
C18:2	54.9	37.9	34.2
C18:3	5.7	1.6	1.3
Total saturated	16.00	17.79	17.67
Total polyunsaturated	60.6	39.44	35.48

[a] 207 g of casein translates to 180 g (the remaining 13% is primarily water, approximately 0.7% phosphorus and 1% fat). [b] Phosphorus: in 2018sc; a portion of this phosphorus is unavailable because it is bound to phytate. That is not the case in the purified diets. [c] The vitamin premix used in the purified diets contains vitamin C; therefore, the value is shown for the purified diets but not 2018sc. There is no requirement for vitamin C supplementation as rodents can synthesize it. Palmitic acid accounts for about 16% of total fat; Infat Oil has about 50% of total palmitic acid at the *sn-2* position.

Table S2. Growth parameters for the preliminary experiment.

Diet	Control *sn-1,3*, Diet	Infat Oil (BPA)
Weight (g)	256.3 ± 12.3	258.9 ± 19.1
Weight gain (g)	201.9 ± 11.2	203.6 ± 15.4
Liver weight (g)	13.6 ± 0. 93	14.2 ± 1.57
Heart weight (g)	1.4 ± 0.2	1.4 ± 0.2
Lungs weight (g)	2.34 ± 0.5	2.35 ± 0.6
Kidneys weight (g)	3 ± 0.3	3.2 ± 0.3
Humeri length (mm)	24.33 ± 0.36	24.26 ± 0.74
EGP height (mm)	0.37 ± 0.03	0.39 ± 0.04

There were no between-group differences in any of the parameters.

Table S3. Serum chemical analysis from the preliminary experiment.

Parameters	Control *sn-1,3*, Diet	Infat Oil (Beta Palmitate) Diet	Reference Values Male SD Rats
Creatinine (mg/dl)	0.298 ± 0.02	0.29 ± 0.05	0.27–0.65
Urea (mg/dl)	53.24 ± 5.71	51.16 ± 6.33	29.3–59.2
Total protein (g/dl)	6.322 ± 0.19	6.385 ± 0.16	5.92–7.46
Cholesterol (mg/dl)	120.2 ± 18.27	115.72 ± 11.14	79–137
Globulin (g/dl)	1.962 ± 0.13	1.97 ± 0.15	1.69–3.01
Triglycerides (mg/dl)	172.3 ± 37.68	194.36 ± 59.05	21–86
Albumin (g/dl)	4.36 ± 0.14	4.40 ± 0.16	3.93–4.73
SGOT (IU/L)	94.5 ± 12.55	114.45 ± 35.17	57–210
SGPT (IU/L)	39.7 ± 3.53	38.54 ± 7.13	30–106
Alkaline phosphatase (IU/L)	289.1 ± 39.05	279.18 ± 34.51	81–197
Calcium (mg/dl)	14.35 ± 0.5	14.21 ± 0.22	9.92–12.28
Phosphorus (mg/dl)	8.44 ± 0.66	8.27 ± 0.61	8.1–12.1
Na (mmol/L)	147.8 ± 1.03	148.45 ± 2.16	142–147
K (mmol/L)	6.02 ± 0.57	6.46 ± 0.92	5.3–7.3
Chloride (mmol/L)	95 ± 1.76	95.45 ± 1.43	94–101
Leptin (pg/mL)	2897.1 ± 1281	3379 ± 1397.4	[1]
IGF-I (ng/mL)	1684.4 ± 141.5	1640.4 ± 252	[1]

Reference values were provided by AML Ltd.

Table S4. Serum chemical analysis after re-feeding.

Parameters	Control *sn-1,3*, Diet (CD)	Infat Oil (BPA) Diet (IO)	Reference Values Male Sprague-Dawley Rats
Creatinine (mg/dl)	0.23 ± 0.03	0.25 ± 0.02	0.27–0.65
Urea (mg/dl)	35.62 ± 3.42	37.225 ± 4.62	29.3–59.2
Total protein (g/dl)	5.6 ± 0.21	5.63 ± 0.12	5.92–7.46
Cholesterol (mg/dl)	144.25 ± 7.56	137.25 ± 10.52	79–137
Globulin (g/dl)	1.5 ± 0.12	1.54 ± 0.23	1.69–3.01
Triglycerides (mg/dl)	87.75 ± 15.94	72.12 ± 16.35	21–86
Albumin (g/dl)	4.1 ± 0.17	4.08 ± 0.15	3.93–4.73
SGOT (IU/L)	96.25 ± 18.74	98.25 ± 32.24	57–210
SGPT (IU/L)	39.62 ± 4.22	38.37 ± 6.44	30–106
Alkaline phosphatase (IU/L)	279.37 ± 41.61	272.25 ± 34.46	81–197
Calcium (mg/dl)	13.64 ± 0.72	13.41 ± 2.12	9.92–12.28
Phosphorus (mg/dl)	9.08 ± 0.51*	10.4 ± 1.44	8.1–12.1
Na (mmol/L)	144.37 ± 2.99	146 ± 2.42	142–147
K (mmol/L)	7.75 ± 0.47	7.55 ± 0.38	5.3–7.3
Chloride (mmol/L)	98.75 ± 1.77	99.5 ± 1.52	94–101
IGF-I (ng/mL)	868.1± 193.7	894.2± 108.1	[1]
Leptin (pg/mL)	1461 ± 196	1448 ± 434.8	[1]

* $p < 0.05$ vs. IO.

Table S5. Top 20 metabolic genes that were affected in the liver by the position of palmitic acid in the diet.

Gene Symbol	Gene Name	Gene ID	Fold Change (IO/CD)
Nampt	Nicotinamide phosphoribosyl transferase	AY 831728	1.86
Alas1	5'-Aminolevulinate Synthase 1	NM 024484	1.76
Mllt3	Myeloid/Lymphoid or Mixed-Lineage Leukemia; Translocated To, 3	BC 129089	1.74
Ltb	Lymphotoxin beta	NM 212507	1.51
Eif2ak2	Eukaryotic Translation Initiation Factor 2 Alpha Kinase 2	NM 019335	1.51
Hsd17b2	Hydroxysteroid (17-Beta) Dehydrogenase 2	NM 024391	1.5
Plek	Pleckstrin	NM 001025750	1.48
Qtrtd1	Queuine TRNA-Ribosyltransferase Accessory Subunit 2	XM 001067760	1.48
Prcp	Prolylcarboxypeptidase	NM 001106281	1.47
Hmgcr	3-Hydroxy-3-Methylglutaryl-CoA Reductase	NM 013134	1.45
Atp2b2	ATPase Plasma Membrane Ca2+ Transporting 2	NM 012508	−1.53
Fbln5	Fibulin 5	NM 019153	−1.58
Fign	Fidgetin	NM 001106484	−1.58
Sgk2	SGK2, Serine/Threonine Kinase 2	NM 134463	−1.61
Dpep1	Dipeptidase 1	NM 053591	−1.68
Lpl	Lipoprotein Lipase	NM 012598	−1.7
Efna1	Ephrin A1	NM 053599	−1.74
Lox	Lysyl Oxidase	NM 017061	−1.85
Rnf125	Ring Finger Protein 125, E3 Ubiquitin Protein Ligase	NM 001108424	−2.24
Egr1	Early Growth Response 1	NM 012551	−3.38

Metabolism was assigned according to the David website (https://david.ncifcrf.gov/).

References

1. Masarwi, M.; Gabet, Y.; Dolkart, O.; Brosh, T.; Shamir, R.; Phillip, M.; Gat-Yablonski, G. Skeletal effect of casein and whey protein intake during catch-up growth in young male Sprague-Dawley rats. *Br. J. Nutr.* **2016**, *116*, 59–69.
